# New Drugs, Appliances, and Things Medical

**Published:** 1892-08-13

**Authors:** 


### NEW DRUGS, APPLIANCES, AND THINGS MEDICAL.

### SANITAS.

Some years ago, when the writer was a demonstrator of anatomy, he was much troubled to find out something which would remove the offensive odour of his hands, which remained even after as many as six or seven washings with soap in hot and cold water. He tried numerous deodorants, and many for a time did away with the smell of adipocere which was so persistent. But after a while the deodorant evaporated, and the sickly, faint odour of decayed humanity reasserted its sway, and the writer went about with the persistent reminder of the post mortem and dissecting rooms always offending his nose. One day, when very fatigued, and consequently more aware of the smell, he happened to glance at a shelf which had become a limbo of forgotten medical and surgical samples, which the generous chemical trade of the country had sent him for trial, amongst these relics of absolute cures and infallible specifics he noticed a dust-grimed bottle labelled "Sanitas Oil." Such was his distrust that he half determined not to try it, but a whiff from his fingers induced him to draw the cork. A little of the oil was well rubbed on the hands and then washed off again with soap and water. The utmost that was expected was that as before, when the disinfectant had evaporated, the same nauseous faint smell would remain. To his surprise and pleasure, after the deodorant had become dissipated, the hands remained sweet, and neither smell of disinfectant nor yet of decay remained. During the two years that he was engaged in teaching anatomy and performing post mortems, Sanitas oil was invariably used after work with invariable success. Notwithstanding this absolute proof of success, such was the power of tradition and prejudice, that it was not till the proprietors and manufacturers showed conclusively, through the labours of an experienced and reliable bacteriologist, that Sanitas was a germ destroyer as well as a deodorant, could the writer come to use it and its derivatives with confidence. He has never found that confidence misplaced, and has at the same time that sense of relieved responsibility which the use of a nonpoisonous remedy implies.

Now what is Sanitas? This requires but a short answer. The essential principles of Sanitas are peroxide of hydrogen and those forms of the camphor group which are produced when the turpene group is acted upon by air and water at summer temperatures. These, like the peroxide, have the power of oxidising and destroying organic substances and low forms of life when they come into contact with them. In addition these latter turpene derivatives are distinctly fragrant and refreshing in odour.

Having found your antiseptic agent, the next thing is to find how to use it with the greatest advantage. It is seldom that one form will answer all purposes, and so it is with Sanitas. Mr. Kingzett, the inventor, has devised many forms, but all containing the active ingredients. First, then, we have the original Sanitas fluid, which is for general disinfecting purposes; it is colourless, non-poisonous, and non-staining. Then there is its crude form, for the larger purpose of street watering, sewers, closets, and so forth. Then oils, which may be used as inhalants in throat and pulmonary affections, and which also are used to charge the inhalers, disinfectors, and disinfecting fumigators. Where heat and light are used a special non-inflammable oil is provided. The disinfecting fumigator is an ingenious block tin arrangement, by which Sanitas oil is volatilized, and the vapours can be inhaled. Where the fumigator is used for disinfecting purposes the non-inflammable oil is recommended to be used. We gave a notice some time back of the Sanitas Disinfector. It is the best apparatus for continued disinfection in foul places that we know of, and we have recommended its use repeatedly for ash-bins during the hot weather, and with great success. In addition a little of the powder should be sprinkled over the offensive waste. The inhaler is a simple and inexpensive apparatus for the application to the throat of the mild and balsamic vapours of the pine products. It is charged with sawdust soaked with the oil. Then for kennels, markets, cattle sheds, poultry houses, and the like, the air purifier and disinfecting sawdust may be used with excellent effects. We can speak highly of these from personal experience. Of the soaps we can but say that their name is legion, from the hard household to the most delicate toilet, and also animal soft soap. This latter is a capital flea destroyer in dogs, very safe as we have tried it. Then the company make insecticide and liquid soap for garden and greenhouse purposes. With a sample sent to us we recently caused an invasion of caterpillars to retreat in greatly diminished numbers. A sheep dip and veterinary ointment complete the list of animal applications. Next we come to a toilet fluid; this is pleasant and active, we find it removes the smell of smoking quickly. Then a well-made tooth powder and a capital nursery powder bring to a close the domestic list. A disinfecting jelly for dressing wounds and skin eruptions, and also for obstetric purposes has been largely tried with success; this with gauze and absorbent wool sum up the surgical appliances made with the Sanitas compounds. In addition, we may mention, a disinfecting furniture cream for general use, or specially for furniture in a room which has been used in infectious cases. Also urinal tablets; these are excellent, and can also be used in the cisterns which supply the closets; a specially designed holder is sold to prevent clogging of the pipes. In conclusion we may add that the company supply peroxide of hydrogen and various other antiseptics, which the knowledge and chemical skill of Mr. Kingzett, their director, have produced. There is no doubt that a non-poisonous disinfectant for general use, *which has been proved to be a germicide*, is to be preferred to one which cannot be safely trusted in non-skilled hands, and this we have in Sanitas and its compounds.

